# The association between doctors’ presenteeism and job burnout: a cross-sectional survey study in China

**DOI:** 10.1186/s12913-020-05593-9

**Published:** 2020-08-03

**Authors:** Pei Pei, Guohua Lin, Gaojie Li, Yifan Zhu, Xiaoyu Xi

**Affiliations:** The Research Center of National Drug Policy and Ecosystem, China Pharmaceutical University, Nanjing, China

**Keywords:** Presenteeism, Job burnout, Emotional exhaustion, Cynicism, Doctors

## Abstract

**Background:**

It is necessary to examine doctors working with illness from a professional point of view, because it is not only related to their occupational health, but more importantly, will affect the treatment effect of patients and the overall medical level of the hospital. The purpose of this study was to explore the relationship between doctors’ presenteeism and job burnout, and to identify other factors that are associated with presenteeism.

**Methods:**

A cross-sectional survey involving doctors (except for primary doctors) was conducted in China. Using one item measure about presenteeism and a 15-item Chinese version of the BMI-GS questionnaire, this study investigated prevalence of doctors’ presenteeism and job burnout, and determined the relationship between presenteeism and job burnout by logistical model.

**Results:**

Relationship between presenteeism and job burnout were explored, and the influence of work factors were evaluated. The survey was completed by 1376/1547 hospital doctors, with a response rate of 88.9%. Presenteeism was reported by 30.7% of participants. Using MBI-GS, 86.8% of all doctors had moderate job burnout and 6.0%(*n* = 82) were severe job burnout. Logistic regression analysis showed that doctors with medium, high degree of emotional exhaustion and high degree of cynicism were more likely to practice presenteeism (all *p* < 0.05). In addition, two other work-related factors, including the doctors’ department and position, were also likely to relate with presenteeism (all *p* < 0.05).

**Conclusions:**

By examining the relationship between presenteeism and job burnout, this study determined that there is indeed a significant correlation between the two. This result has a certain reference value for the development of work health, especially presenteeism and job burnout theory, and also makes a certain contribution to the relevant research literature.

## Background

Presenteeism refers to the behavior of employees attending work while ill, widely considered a negative phenomenon at present [1]. There are two main understandings of presenteeism. One is represented by American medical scholars and health consultants, who believe that presenteeism refers to loss of productivity due to individual health problems [2–6]. The second understanding is represented by European scholars, who believe that presenteeism refers to working despite illness. Their research mainly focuses on why employees make the decision to work with illness [7–12]. In contrast to the former, the latter limits the definition of presenteeism to the behavioral level and avoids the confusion of causal relationship of presenteeism [13]. In our view, relevant research should shed more light on the act of presenteeism.

Presenteeism in various occupations has been investigated in the United States [14], Canada [15], the United Kingdom [16], South Korea [17] and other countries, and the prevalence of this behavior varies from 30 to 90%. Compared with other professional groups, doctors have a higher prevalence of presenteeism [10, 18, 19]. Moreover, when doctors contract contagious diseases or other illnesses, they negatively affect the health of patients and other medical workers as well as their own health [8, 15, 20], which causes a significant impact and serious consequences to the patient population and the medical system [21, 22]. In practice, however, attention to doctors’ presenteeism is still relatively insufficient, at the same moment, the study concerning presenteeism needs further updates and additions.

Actually, many countries, including China, have already conducted researches on presenteeism, and found that there are some significant factors related to presenteeism, including depression [23, 24], anxiety [23, 24], pressure [25], stress [26], and physical disease, such as bone pain and muscle soreness [27]. In addition, with high prevalence of presenteeism, symptoms of job burnout have also been frequently reported especially among doctors [28–31]. However, the research on the correlation between presenteeism and job burnout is less than the aforementioned factors.

There are many definitions of job burnout, among which Maslach’s three-dimensional concept of job burnout is widely accepted [32]. Maslach suggests that job burnout is a syndrome of emotional exhaustion, cynicism and reduced personal accomplishment in the process of daily contact with clients [33]. Lotta D et al. conducted a cohort study involving medical personnel and found that presenteeism was an important risk indicator to job burnout, health status, and sick leave, but did not specify how these factors interact with [34]. After systematically reviewed the published literature, it is further clarified that presenteeism is the occupational result of job burnout [35], but no practical research was taken out to prove this argument. Only a three-wave study of in-patient nurses pointed out by practice that the depersonalization is the result of long-term presenteeism, and emotional exhaustion and presenteeism are reciprocal [36]. Therefore, to explore the correlation between presenteeism and job burnout, more scientific methods and rigorous analysis are required.

In the past, some scholars had proposed many theoretical models when discussing job burnout. Among them, Job Demand-Resource Model [37] and Maslach General Model of Burnout [32, 33] are two classic theoretical models. The Job Demand-Resource Model shows that when job demands are high and job resources are low, the risk of job burnout is higher [37]. Job demands are organizational, social or physical aspects of the job that require sustained physical and/or psychological effort from the employee, according to which, presenteeism may constitute a demand that can have an effect on employees’ health and well-being [37]. Proposed by Maslach and Leiter in 1996 [32], the General Model of Burnout is similar to the Job Demand-Resource Model, and it is believed that uncoordinated job demands and resources can lead to burnout. The final possible outcomes of burnout include absenteeism, turnover, physical/mental illness, and a decline in organizational commitment, and so on. Based on the above, it is assumed that whether presenteeism is regarded as a kind of job demand or as one of the possible results of job burnout, presenteeism is likely to have a great connection with job burnout.

The high prevalence of presenteeism among doctors highlights the importance of systematic investigation of this phenomenon, because it may affect the medical treatment effect of patients [10, 26], the quality of doctors’ work [2, 26] and their long-term health [2]. The study on the relationship between presenteeism and job burnout and presenteeism-related factors is of great significance for establishing appropriate job design theory and occupational stress intervention measures, thus reducing the prevalence of medical job burnout and presenteeism. Therefore, this research plan understands the actual level of Chinese doctors’ presenteeism and job burnout, and refines each dimension of job burnout to conduct specific analysis to clarify the correlation between presenteeism and job burnout.

## Method

### Study design and sampling

Based on informed consent and voluntary participation, a cross-sectional study was conducted from July to August 2019 to investigate the status of presenteeism and job burnout among medical practitioners in China’s second- and third-class medical institutions. In China, medical institutions are divided into three levels: The first class is small primary medical institutions; the second class is medium-sized medical institutions; the third is large tertiary medical institutions. The functions of these different levels of medical institutions are different. The second- and the third-class medical institutions, which are mostly municipal, provincial or national general hospitals, provide specialized health protection and nursing services for many regions and play important roles in medical education and scientific research. Some studies have found that the incidence of presenteeism in primary medical institutions is slightly lower [38, 39] than in the second- and the third-class medical institutions. Therefore, for greater representativeness, doctors working in second- and third-class institutions were selected as the research object.

To make the sample coverage more extensive and reflect the overall working status of Chinese doctors, a multistage sampling method was adopted in this study. The steps are as follows.

(1) All 31 provinces (autonomous regions and municipalities directly under the Central Government) in mainland China were included in the sampling. In each province, all cities/urban areas under provincial jurisdiction were divided into 3 urban groups (high, medium and low) according to their GDP per capita in 2018, for a total of 93 urban groups in 31 provinces.

(2) Among hospitals permission was granted to conduct the investigation, hospitals were selected by convenience sampling. At least 2 s-class hospitals and 2 third-class hospitals were selected from each city group.

(3) In each hospital in which at least two doctors were willing to participate in and complete the investigation, we used convenience sampling to select the interviewees. The criteria for the selection of doctors were outpatient or inpatient doctors who had obtained medical qualification certificates, held positions in clinical departments or had prescription rights. Doctors excluded from the selection were nonclinical doctors, such as laboratory technicians, nonprescription doctors (such as doctor assistants) and doctors who were not registered in the sample hospital.

### Questionnaire design

The questionnaire was categorized into three parts. The first part was a series of questions about demographic and work-related information suggested to be associated with presenteeism [40]. Among these demographic factors, age, sex, marital status, number of children and educational level were measured [40]. Working factors mainly included hospital department, working years, professional title and position [7, 40, 41].

The second part followed Aronsson’s suggestion and used a single question to evaluate doctors’ presenteeism [19]. The dependent variable of presenteeism was measured by asking the doctor “the number of times in the past year (12 months) that he or she had to take time off for physical reasons but still had to work”. The answers were “never”, “once”, “2–5 times” or “more than 5 times”. The measurement method has high internal consistency when used in Chinese population, and the retest reliability is 0.31 [42]. The response scale was dichotomized for the purpose of logistic regression (0 = never/once, 1 = 2–5 times/ more than 5 times) [13, 19, 40, 43].

The third part was a general scale of job burnout, which was adopted to capture the level of doctors’ job burnout. The Chinese version of the 15-item Maslach Burnout Service Inventory (MBI-GS) was adopted, the usage permission of which has been obtained through official channels. In 2002, Chaoping Li carried out exploratory factor analysis on 16 items of MBI-GS, using principal component method to extract factors and orthogonal rotation axis, and found that one item of “cynicism” had high cross load. After deleting this item, the factor analysis was carried out again. The adjusted MBI-GS structure is completely consistent with the original, which shows that MBI-GS has good conceptual validity in China. The internal consistency coefficients of emotional exhaustion, cynicism and reduced personal accomplishment are 0.88, 0.83 and 0.82 respectively [44].. The questionnaire uses 7-point Likert scale, with 0 representing “never” and 6 representing “very frequent”, including 5 questions on emotional exhaustion, 4 questions on cynicism and 6 questions on personal accomplishment. A total score of emotional exhaustion greater than 14 indicates high degree of emotional exhaustion, while a total score of emotional exhaustion less than 11 indicates low degree [45]. A total score of cynicism higher than 7 indicates the existence of obvious emotional alienation, while a total score of cynicism lower than 5 indicates low degree of cynicism [45]. A score of over 29 in personal accomplishment indicates low sense of achievement, while a score of less than 26 indicates high sense of achievement [45]. The total score of job burnout = 0.4 × emotional exhaustion average+ 0.3 × cynicism average+ 0.3× personal accomplishment average. Total scores that fall into the ranges of 0 ~ 1.49, 1.50 ~ 3.49 and 3.50 ~ 6 indicate the conditions of no burnout, moderate burnout and severe burnout, respectively [46].

### Data collection

This study selected undergraduates who had received research and training and had professional medical backgrounds as investigators. Each department had at least 5 doctors who agreed to participate in the survey. The doctors in the sample hospital were interviewed randomly, and relevant information was collected. The content of training for the researchers included the background, purpose, etiquette, methods and skills of the research, methods of dealing with emergencies and the use of research software purchased and redeveloped by the research team.

The content of this study was examined by the Ethics Review Committee of China Pharmaceutical University. Before the formal investigation, the researcher identified the nonworking hours of the hospital and entered the hospital with the oral permission of the hospital director/deputy director. The researcher orally introduced the background, content and purpose of the investigation to the doctor and started the questionnaire for those who were willing to participate in the investigation and sign the informed consent. During the investigation, the researcher opened the questionnaire with the research software in a mobile electronic device, explained the requirements for answering questions in detail, read the questionnaire items and the answers to the multiple-choice questions aloud, and recorded the oral responses of the respondents with the software. The results of the pre-survey show that this interview method is feasible and the reliability and validity of the survey are high. Moreover, the questionnaire is relatively short, and the interviewees will not be tired and agitated, so that the interviewees can answer seriously and better avoid too casual data. All the research was conducted in an undisturbed environment, and the researcher did not show their opinions or any inclinations regarding the research content before or during the research [47].

### Data analysis

Presenteeism was the dependent variable, and job burnout was the independent variable. The control variables included demographic and working factors, and descriptive statistics were performed on all data collected from the doctors surveyed, as detailed in Table 1. This study employed a binary logit model to explore the relationship between presenteeism and job burnout. Logistic regression analysis was performed using STATA13.0-SE software, with *P* < 0.05 as the level of significance.

**Table 1:** Prevalence of presenteeism, job burnout among the participants (*n* = 1376)

Prevalence of presenteeism		Never or once	Twice or more	Sum
Degree of job burnout				
Emotional exhaustion	Low(≤10)	47.67%	15.41%	63.08%
	Medium(11–14)	12.50%	8.79%	21.29%
	High(≥15)	9.16%	6.47%	15.63%
Cynicism	Low(≤4)	43.53%	14.39%	57.92%
	Medium(5–7)	16.06%	7.34%	23.40%
	High(≥8)	9.74%	8.94%	18.68%
Personal accomplishment	Low(≥30)	20.86%	9.96%	30.81%
	Medium(26–29)	7.70%	2.98%	10.68%
	High(≤25)	40.77%	17.73%	58.50%
Total (=0.4*EE+ 0.3*Cy + 0.3*PA)	No(≤1.49)	6.03%	1.16%	7.19%
	Moderate(1.5–3.49)	60.32%	26.53%	86.85%
	Severe(≥3.5)	2.98%	2.98%	5.96%
Sum		69.33%	30.67%	

## Results

A total of 1376 doctors from 305 third-class hospitals and 349 s-class hospitals participated in the survey, of whom 46.9% were women, 82.6% were married, most were aged 25–44 (70.8%), 65.0% had a master’s degree and 26.9% were internal medicine doctors, 55% had worked for more than 10 years, and more than 70% had junior or middle professional titles.

Table 1 shows the frequency of doctor presenteeism and job burnout in this study. Of the 1376 doctors, 30.67% (*n* = 422) reported that they had worked when they were not feeling well twice or more over the previous 12 months. Regarding job burnout, 86.85% (*n* = 1195) of the doctors indicated moderate burnout, and 6.0% (*n* = 82) of the doctors indicated severe burnout. Specifically, 15.63% (*n* = 215) of the doctors reported significant emotional exhaustion at work, 18.68% (*n* = 257) reported cynicism about work, and 58.50% (*n* = 805) reported reduced personal accomplishment. Among the doctors who confirmed job burnout, the proportion of doctors who had practiced presenteeism was 29.51% (*n* = 365), which was almost close to the proportion (30.67%) among all the surveyed.

### Regression analysis

With the three dimensions of job burnout as three independent variables, the logistic regression analysis showed that there were significant differences among those variables related to presenteeism, including two dimensions of job burnout, emotional exhaustion and cynicism, as shown in Table 2. Compared with doctors with low degree of emotional exhaustion or cynicism, doctors with medium, high degree of emotional exhaustion or high degree of cynicism were more likely to practice presenteeism twice or more (all *p* < 0.05). In addition, two other work-related factors, including the doctor’s department (Emergency) and the doctor’s position (Deputy chief doctor), were more likely to be associated with their presenteeism behavior, which were controlled in our regression analysis (all *p* < 0.05).

**Table 2:** Logistic regression analysis of factors associated with presenteeism

Items	Odds ratio	*P*	95% confidence interval
Medium degree of emotional exhaustion	1.830	***0.000***	1.325–2.526
High degree of emotional exhaustion	1.527	***0.030***	1.041–2.240
Medium degree of cynicism	1.245	0.197	0.893–1.735
High degree of cynicism	2.421	***0.000***	1.664–3.522
Medium degree of reduced personal accomplishment	0.843	0.464	0.533–1.333
High degree of reduced personal accomplishment	0.811	0.164	0.603–1.090

## Discussion

The main results of this study confirm our initial inference that when we control the known factors related to presenteeism, presenteeism is related to two dimensions of job burnout, namely emotional exhaustion and cynicism. Earlier, Ferreira A. I. et al. studied the correlation between job burnout and presenteeism of 281 junior high school teachers in Portugal [48]. They believed that presenteeism was related to the three dimensions of job burnout, and the correlation coefficients of emotional exhaustion, cynicism and personal accomplishment were 0.238, 0.166 and-0.118 respectively [48]. According to Demerouti and her colleagues, an employee, present but sick could become a more exhausted employee [36]. Accordingly, employees who experienced compensatory exhaustion-activating strategies like sickness presenteeism, could in turn increase their exhaustion. Another perspective was put forward by De Vroome E. et al. [49], who believed that emotional exhaustion may be an important determinant of presenteeism. Demerouti also agreed that it was likely that sickness presenteeism and burnout had reciprocal relationship [36]. These show that job burnout and presenteeism do have a correlation that cannot be ignored, and especially that the relationship between emotional exhaustion and presenteeism has been confirmed many times.

According to the job demand-resource model [37] and the general model of job burnout [32, 33], higher job demands and lower job resources are the important causes of job burnout, according to which, presenteeism may constitute a demand that can have an effect on employees’ health and well-being [37]. For doctors, especially those in the second-and third-class hospitals, social support is of great significance to the health and well-being of them [50]. According to the labor force statistics of doctors in 2012, there were only 1.6 doctors per 1000 people in China, with the number of doctors per capita severely below the OECD’s average of 3.2. The growing demand for medical services has led to a sharp increase in doctors’ workloads. Given the overwhelming pressure of medical needs, doctors are pushed to work with illness, even when they have job burnout at the same time (the proportion of these doctors is 29.51% in our study), thus tensions between doctors and patients have often been exacerbated. Medical disputes, deteriorating relations between doctors and patients, and even violent attacks on doctors have become serious problems in China [51–53].

Apart from the shortage of medical resources, there are many reasons why doctors choose to work despite illness. As Aronsson and Gustafsson explained in their conceptual model [7], this issue involves a very complex decision-making process for doctors. Contributing factors include personal needs and work-related needs. In our study, two work-related factors are mainly involved in the control variables. Specifically, emergency doctors and doctors with managerial responsibilities are more likely to show presenteeism. It is necessary to pay more attention to the health status of doctors with more stress and more complex work tasks to reduce the prevalence of presenteeism. At the same time, short-term leave for doctors should be granted, which may help doctors to make physical and mental adjustments themselves. Soler et al. found a significant relationship between job burnout and sick leave [54], while Siu et al. did not [55]. That’s probably because the type of decline in productivity demonstrated by doctors experiencing job burnout may be related to their environment. In other words, sick leave may be allowed in one system but discouraged in another. A system that shows less tolerance toward sick leave will inevitably inspire doctors’ presenteeism.

### Limitations

This study has some limitations. First, the 12-month recollection period for participants regarding presenteeism may be excessively long, which may lead to inaccurate self-reports by doctors. Second, we used a self-rating scale to measure doctors’ presenteeism and job burnout, which should be evaluated more objectively in future studies. Third, the design was a cross-sectional survey and mainly focused on the correlation between presenteeism and job burnout, but it did not mean a hint about the causality between them. Finally, the subjects in this study were doctors in the second- and third-class hospitals, excluding primary doctors. Future studies on doctors working in different environments could enhance the understanding of doctor presenteeism.

## Conclusion

The results of this study have certain practical application value. This study has a certain reference value to the development of work health, especially presenteeism and occupational burnout theory by examining the relationship between presenteeism and employee burnout. In earlier studies, health problems have been linked to job burnout, but more often as a result variable or the consequence of burnout. In this study, presenteeism is significantly associated with job burnout when known factors such as individuals and work-related factors is controlled. Therefore, these findings not only contribute to the literature about burnout, but also to the wider research on presenteeism. At present, the working environment of many employees is characterized by high requirements, responsibilities, and workload. And such a working environment often produces contradictions and conflicts. Although the current research focuses on doctors in senior medical institutions, we believe that the current research results can be considered applicable to multiple occupations.

## Data Availability

The datasets used and/or analyzed during the current study are available from the corresponding author on reasonable request.

## Abbreviations

MBI-GS: Maslach Burnout Inventory-General Survey
GDP: Gross Domestic Product
EE: Emotional exhaustion
Cy: Cynicism
PA: Personal accomplishment
OECD: Organization for Economic Co-operation and Development
